# Case Report: Post-stroke hemorrhagic infarction in a status epilepticus Beagle dog

**DOI:** 10.3389/fvets.2026.1764817

**Published:** 2026-03-25

**Authors:** Nikita M. Joshi, Raghul Jayaprakash, Pratik P. Panchal, Urvit P. Patel, Ramchandra Ranvir, Satish Patel, Rajesh Sundar, Mukul R. Jain

**Affiliations:** 1 Department of Pharmacology and Toxicology, Zydus Research Centre, Zydus Lifesciences Limited, Ahmedabad, India; 2 Canine Research Facility, Zydus Research Centre, Zydus Lifesciences Limited, Ahmedabad, India

**Keywords:** angiogenesis, Beagle, epilepsy, extracellular matrix, post-stroke epilepsy, seizure, status epilepticus, VEGF

## Abstract

This case describes the acute neuropathology of a 4.5-year-old male Beagle that presented with status epilepticus and a simultaneous post-stroke hemorrhagic infarction. The clinical course involved 5 days of refractory cluster and generalized seizures, during which the patient remained in a “grave” borderline threshold on the Modified Glasgow Coma Scale. Initial hematobiochemical analysis revealed marked elevated levels of serum creatine kinase and aspartate aminotransferase, indicating severe muscular exertion, alongside dehydration and a 30% decline in serum albumin levels. Concurrent absolute monocytosis and neutropenia were observed, reflecting a systemic neuroinflammatory response. Treatment included intravenous diazepam boluses followed by a constant rate infusion of oral potassium bromide and hypertonic saline to manage suspected cerebral edema; however, the animal remained refractory to therapy. A gross examination at necropsy showed that the frontal lobe had multifocal tarry red subarachnoid and parenchymal hemorrhage extended to the olfactory bulb with a contralateral dull gray focus of infarction. Histopathological analysis confirmed multifocal thrombosis and liquefactive necro-hemorrhagic foci, accompanied by significant neuronal degeneration and spongiosis. The newly formed vessels in the peri-infarct regions demonstrated robust expression of VEGF-A and reticulin, which is an early marker of inflammatory extracellular matrix changes, while mature collagen and elastin were completely absent. These findings indicate that the observed vascular proliferation was an acute and active process rather than a pre-existing lesion, contrasting with previous findings on chronic epilepsy in dogs. This case reinforces the critical role of VEGF-A-driven angiogenesis and vascular growth in the development of acute structural epilepsy in dogs. Furthermore, the case exhibits features of post-stroke epilepsy, indicating a potential tissue-based marker that could enhance the detection of seizure recurrence through advanced imaging and assist in the discovery of novel therapeutic targets.

## 1 Introduction

Epilepsy is one of the most prevalent chronic neurological disorders in dogs, affecting an estimated 0.5–5% of the general canine population and accounting for 1–2% of cases in referral veterinary hospitals (1). Among canine breeds, the Beagle has emerged as a particularly valuable model for epileptogenic research due to its extensive use in laboratory experiments and its well-documented genetic predisposition to the condition. To date, more than 50 epilepsy-associated mutations involving ion channels and neurotransmitter receptors have been identified in this breed. Furthermore, the biometric characteristics of genetic epilepsy in Beagles include a significant sire effect, a male preponderance, and a clinical onset that can occur as early as 1 year and up to 7.35 years, with a median onset age of 5.71 years (2, 3).

According to canine epidemiological data, when the underlying cause of genetic epilepsy remains unidentified, it is classified as idiopathic or of unknown origin, whereas identified causes are categorized as structural epilepsy, and both groups account for approximately 63 and 37% of cases, respectively (4). Regardless of the specific type, status epilepticus (SE) and severe cluster seizures occur at a higher frequency than other seizure types, such as isolated or focal seizures (5). In this report, we present a borderline case involving a Beagle dog with a known genetic predisposition who also exhibited acute structural brain lesions. While a pre-existing structural cause for the initial SE was not identified, we found acute stroke-related lesions that appear to have triggered the subsequent seizures.

In dogs, stroke is often secondary to conditions such as hypothyroidism, neoplasia, sepsis, systemic hypertension, parasitic migration, vascular malformations, or coagulopathy (6). Brain infarction, a common sequela to stroke, is most frequently observed in dogs with concurrent chronic kidney disease or hyperadrenocorticism, as documented in magnetic resonance imaging (MRI) studies (7). Furthermore, dogs with hyperadrenocorticism and hypercoagulability are prone to thromboembolism, whereas children with prolonged status epilepticus have been shown to develop disseminated intravascular coagulation (8, 9). In addition to these vascular events, neurogenic myocardial stunning attributed to catecholamine-mediated autonomic dysregulation has also been reported in canine patients (10).

While ischemic infarction is a typical feature of thrombotic events associated with status epilepticus, our case revealed a more severe and less frequently reported form—hemorrhagic infarction. The clinical progression observed in this animal was comparable to post-stroke epilepsy (PSE). In human medicine, PSE is classified based on the timing of recurrence: early (occurring within 4–7 days) or late (exceeding 7 days). The development of epileptogenic tissue following a stroke provides an opportunity to determine the duration of seizure recurrence through advanced imaging and to explore novel therapeutic interventions (11, 12). Currently, targeted anti-epileptogenic drugs for PSE are unavailable, and animal models suggest that an active epileptic focus is central to its origin. These foci are considered to arise from post-stroke neuroinflammation, blood–brain barrier (BBB) disruption, and maladaptive neuroplasticity.

The primary aim of this investigation was to identify and characterize the morphological focus of post-stroke epilepsy in a dog that experienced refractory seizures until its moribund sacrifice. Second, we examined this focus for evidence of pre-existing lesions, comparing our findings to established post-mortem markers of chronic epilepsy in dogs. Specifically, we evaluated the glial response and endothelial proliferation to assess the chronicity of the condition and the process of epileptogenesis (13, 14). We utilized vascular endothelial growth factor (VEGF) expression as a marker for epileptogenesis, as it is a hypoxia-driven event previously validated in beagle pup models (4). Consequently, this study uses VEGF immunofluorescence and extracellular matrix (ECM) special staining, which is analyzed in conjunction with clinical history and histopathological findings to further our understanding of acute structural epilepsy.

## 2 Case description

### 2.1 Case presentation

A 4.5-year-old male Beagle from a canine research colony breeding stock presented with an acute onset of seizures characterized by sudden episodes. Prior to this occurrence, the animal’s clinical history and observations were unremarkable. The dog experienced generalized cluster seizures that rapidly progressed to status epilepticus (SE). Clinical signs during the SE phase included hyperextended limbs without opisthotonos, foamy salivation, conjunctival hyperemia, and pyrexia (104.2 °F). The entire seizure episode spanned 5 days (Supplementary Table S1).

Initial hematobiochemical analysis revealed elevated levels of creatine kinase (CK) and aspartate aminotransferase (AST), indicating severe muscular exertion, alongside dehydration indicated by increased blood urea nitrogen (BUN) and creatinine levels. Serum sodium and potassium levels were also at the upper limit of the reference range (Supplementary Table S2). To stabilize the animal, an intravenous bolus of diazepam (Abbott Healthcare Pvt. Ltd., Mumbai) was administered at 1 mg/kg, followed by a continuous infusion to manage recurrent seizures. On day 2, a loading dose of oral potassium bromide (Rasayan Trading Co, Ahmedabad) was initiated for SE management; however, administration could not be sustained due to persistent jerky jaw movements that complicated intubation (Supplementary Figure S3). The patient was maintained on a normal saline-dextrose infusion and received intensive supportive care, including the use of foam bedding and an elevated head posture.

The clinical diagnosis was primarily based on neurological signs and hematological data. Advanced diagnostics, such as magnetic resonance imaging (MRI) or cerebrospinal fluid (CSF) analysis, were precluded due to the animal’s unstable condition. While the initial hypoglycemia on day 0 suggested a reactive seizure or idiopathic epilepsy, the persistent lack of consciousness during inter-ictal intervals indicated underlying structural brain changes. Throughout the episode, the patient remained in lateral recumbency. Based on the Modified Glasgow Coma Scale (MGCS), scores for consciousness and reflex activity remained within the “grave” borderline threshold. Due to the lack of response to stimuli and suspected cerebral edema, 7.5% hypertonic saline was administered.

By day 4, the animal had become comatose and refractory to treatment. Following institutional animal welfare protocols, euthanasia was performed via an overdose of thiopental sodium. On the day of euthanasia, hematobiochemical profiles showed extremely elevated levels of CK and monocytes, reflecting ongoing necrosis and neuroinflammation. Additional terminal findings included significantly low albumin levels, critically low neutrophil counts likely due to severe stress, and a marked decline in platelet counts from 490 to 290 × 10^3^/μL. An increased blood urea nitrogen/ creatinine ratio implies non-renal and systemic catabolism.

### 2.2 Investigation

A complete necropsy was conducted, and the brain was examined *in situ* before being carefully removed and fixed intact via immersion in 10% neutral buffered formalin (NBF) for 72 h. Other major organs, including the spinal cord, lungs, liver, kidneys, heart, spleen, skeletal muscle, and sciatic nerve, were also collected and fixed in 10% NBF. Following fixation, the brain was sectioned coronally according to a detailed, standardized Tier II neuropathology protocol (15). Representative samples from the affected frontal lobe and the unaffected control regions were processed for paraffin embedding.

For routine histopathological evaluation, 4–5 μm sections were stained with Harris hematoxylin and eosin (H&E). Special stains, including modified Verhoeff–Van Gieson, James’ silver stain, and Masson’s trichrome, were applied to adjacent sections to evaluate elastin, reticulin, and collagen, respectively. Additionally, the deparaffinized sections underwent immunofluorescence staining using a canine-specific primary antibody (mouse anti-VEGF-A, GeneTex, GTX21316, 1:20) and a secondary antibody (goat anti-mouse IgG conjugated to Alexa Fluor™ 488, Invitrogen, 1:200), with 4′,6-diamidino-2-phenylindole (DAPI) used as a nuclear counterstain. All sections were evaluated using a Nikon ECLIPSE 80i microscope, and images were captured with NIS-Elements AR 4.3 software.

**Figure 1:**
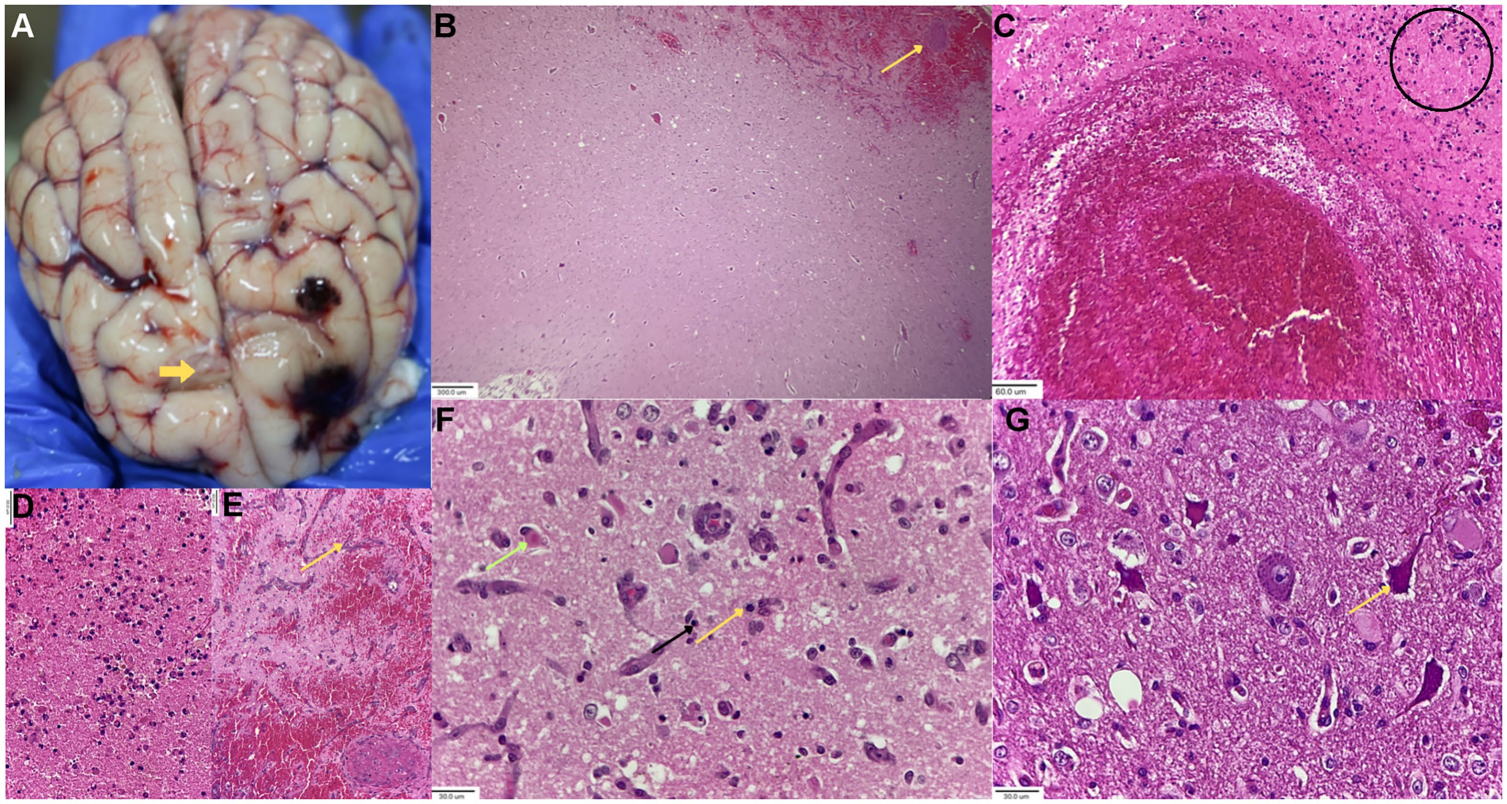
Representative gross and histopathological hematoxylin & eosin-stained images of the epileptic dog brain. **(A)** Gross multi-focal hemorrhages in the frontal cortex and olfactory lobe in the left hemisphere, and a dull area of focal necrosis (arrow). **(B)** 4× magnification of endothelial proliferation beneath a superficial cortical hemorrhage and thrombus (arrow). Thrombus shown in Panel **(E)**. **(C)** Necrohemorrhagic centric lesion in cerebral cortex at 20× magnification. Encircled area is shown at higher magnification in **(D)**. **(D)** 40× magnification of circle in Panel **(F)** shows dead glial and neuronal cells with PMN infiltrates. **(E)** 40× magnification of thrombus shown in Panel **(B)** with endothelial proliferation (arrow). **(F)** 40x magnification of endothelial proliferation in the periphery of the lesion with mitotic figure (Yellow arrow), an astrocyte (black arrow), and an eosinophilic dead neuron (green arrow) shown. **(G)** 40× magnification of dark, basophilic, shrunken ischemic neurons (arrow) near a hemorrhagic focus area.

Gross examination revealed multifocal, tarry red hemorrhagic foci within the prorean and precruciate gyri of the frontal lobe, extending to the olfactory bulb (Figure 1A). This subarachnoid hemorrhage was focal and confined, but it also extended into the brain parenchyma. Notably, there was no evidence of subcutaneous cranial hemorrhage, cerebral swelling, or depressed surface lesions. A dull gray infarct was identified on the contralateral side of the frontal lobe, adjacent to the frank hemorrhage (Figure 1A; Supplementary Figures S1–S3).

Histopathology confirmed a liquefactive necro-hemorrhagic focus across all coronal slices from the olfactory bulb to the prefrontal lobe. Thrombi were identified within the vessels of the hemorrhagic tissue in multiple brain locations (Figure 1B). The center of the hemorrhagic infarct was characterized by significant erythrocyte accumulation, neutrophilic infiltration, and degenerative cellular debris. In contrast, the infarct penumbra exhibited profuse cellular proliferation, multifocal spongiosis, and neuronal death (Figures 1C,D).

The observed proliferation consisted primarily of budding endothelial cells and immature vessels (Figures 1E,F). Degenerated neurons presented as shrunken cells with eosinophilic, ill-defined nuclei (Figures 1F,G), while ischemic neurons in the penumbral zone appeared dark, basophilic, and vacuolated (Figure 1G). These necrotic areas were intermingled with glial cells, including reactive astrocytes, swollen oligodendroglia, and minimally involved microglia (Supplementary Figures S4, S5).

**Figure 2:**
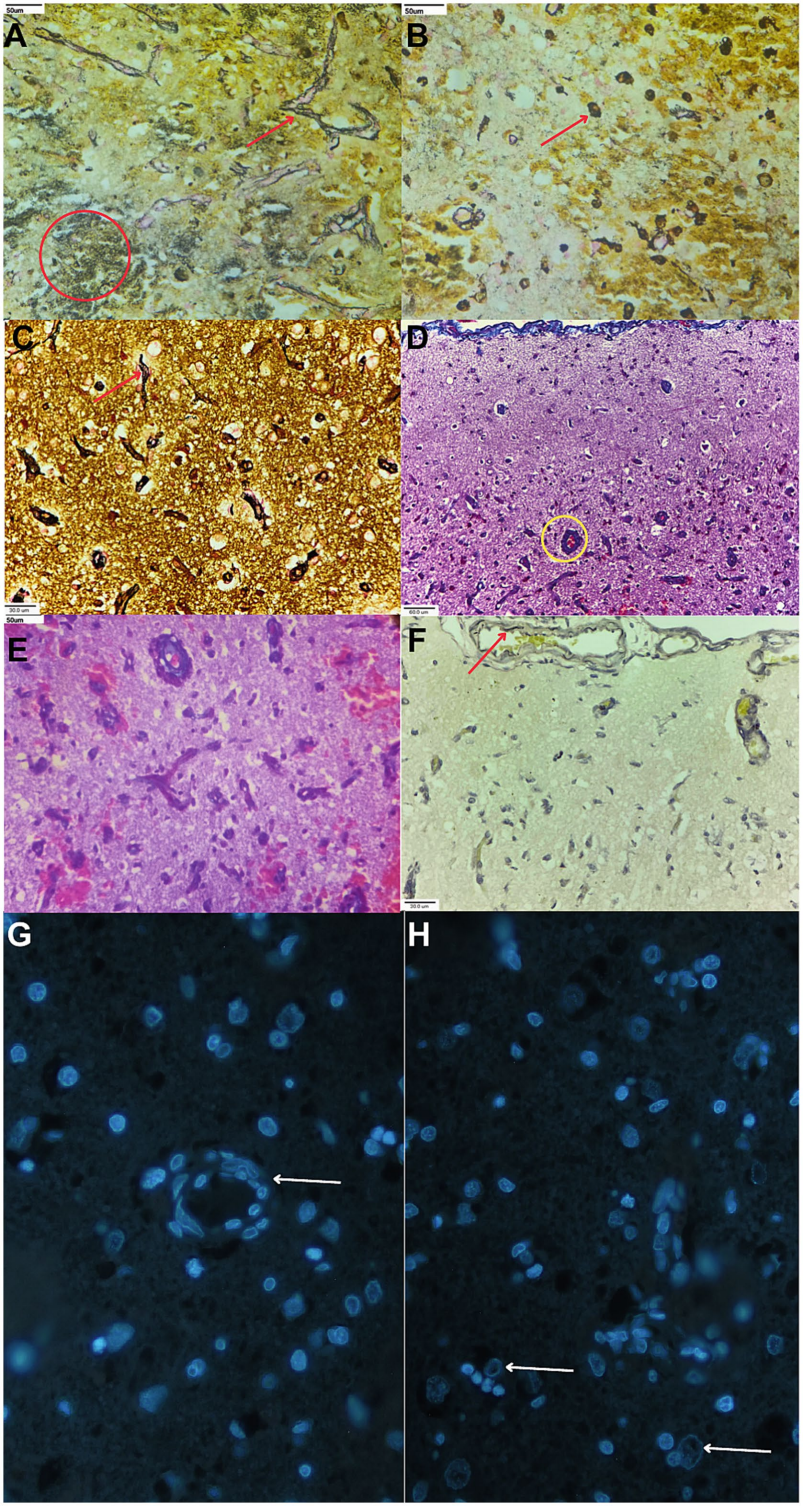
Special staining of the extracellular matrix and immunofluorescence of VEGF and related changes in the epileptic dog brain. **(A)** 40× magnification of reticulin staining with James Silver Stain (JSS) of blood vessels (red arrow) and necrotic areas (circle) from the frontal cortex. **(B)** 40× magnification of strong reticulin staining with JSS of new budding endothelial cells (arrow) in the frontal cortex. **(C)** 40× magnification of a strand of reticulin fibers from a vessel shown (arrow) with JSS in the frontal cortex. **(D)** Weak collagen staining with Masson’s trichrome (MT) in areas of new endothelial growth (circle), shown at 20× magnification. **(E)** 40× magnification of the circled area in Panel **(D)** shows weak blue MT stain. **(F)** Absence of elastin staining with modified Verhoeff–Van Gieson in the areas of neovascularization. Note the contrasting positive elastin staining in the tunica interna of a mature vessel (arrow). **(G)** 100× magnification of a VEGF-A reactive vessel (arrow) from the necro-hemorrhagic lesion shows green fluorescence and cell nuclei with blue fluorescence from 4′,6-diamidino-2-phenylindole (DAPI) counterstain (merged). **(H)** 100× higher magnification of VEGF-A positive, green fluorescing inflammatory cells. The cell membrane shows green fluorescence (arrows) with blue DAPI nuclear counterstain (merged).

Special staining for reticulin was strongly positive in both the central and peripheral regions of the lesion. Conversely, collagen staining was weak and restricted to the periphery (Figures 2A–E). These connective tissue reactions originated from new vascular growth or intervening extracellular matrix components, while elastin staining was entirely negative (Figure 2F). VEGF-A expression, visualized via green fluorescence, was localized along the membranes of newly formed blood vessels and surrounding glial cells (Figures 2G,H; Supplementary Figure S6). No significant lesions were identified in the remaining brain regions or other systemic organs.

## 3 Discussion

This case report describes the clinical, gross, and histopathological features of an epileptic lesion in a dog that experienced a stroke. The analysis of the lesion focuses on the cerebral hemorrhagic infarct accompanied by angiogenesis, highlighting an acute form of canine epilepsy that differentiates it from previously well-documented chronic epileptic brain changes. Additional findings concerning extracellular matrix (ECM) changes and VEGF expression shed light on the early tissue changes associated with post-stroke epilepsy (PSE), which are relevant to the equivalent condition in humans (11).

In dogs, idiopathic epilepsy typically occurs between 6 months and 6 years of age; incidents outside this range often imply a structural cause where the underlying pathology can be identified (1). For our examination of a 4.5-year-old male Beagle, we considered the breed’s male preponderance and its known genetic susceptibility to epilepsy. As a result, idiopathic epilepsy was our initial tentative diagnosis (2). However, structural changes involving a hemorrhagic infarct and significant vascular growth were observed, necessitating further investigation. We gathered clinical signs and other data to infer the possible causes of the infarct. In an extended analysis of epilepsy in dogs (*n* = 254), cluster seizures were found to be common (47.2% incidence), with 24.6% progressing to status epilepticus (SE). SE is characterized by abnormal clinical and neurological findings and often necessitates euthanasia. Of the SE cases, 35.5% are typically classified as structural epilepsy, particularly in the age group approximately 43–139 months (16). In our clinical observation, a possible structural change was evident in the loss of consciousness between seizures. Following initial cluster seizures, this condition progressed to SE. This adversity in SE stems from a catecholamine surge that can induce neurological emergencies such as neurogenic stunned myocardium and sudden unexpected death in epilepsy (SUDEP) (4, 10). Furthermore, high cortisol levels in dogs can damage the brain’s autonomic control areas in cases of neurogenic stunned myocardial condition and may lead to thromboembolism associated with hyperadrenocorticism (8). Similarly, in children experiencing status epilepticus, hyperthermia from continuous muscle activity can cause disseminated intravascular coagulation (9). We observed indications of a vascular crisis in the morbid animal, characterized by a significant decrease in platelet count and the presence of a microscopic thrombus. Additionally, persistent hyperthermia, high creatine kinase and AST levels, and clonic–tonic muscle contractions were noted. Other potential causes, such as pyrexia of viral origin, parasitic migration, or emboli, were ruled out based on the animal’s health and vaccination records (1). The underlying causes of canine stroke, such as chronic kidney disease, heart conditions, Cushing’s syndrome, and hepatic encephalopathy, were not suspected, as recent physical examinations and serum biochemistry values before euthanasia were normal, and histopathology of the kidney, heart, adrenal glands, and liver yielded no related findings.

The gross examination of the animal revealed no signs of edema, such as widening or flattening of the sulci or mild deviations of the falx cerebri, which are the findings commonly associated with canine ischemic stroke. A focus of gray infarct on the contralateral side of the hemorrhagic prefrontal lobe indicated a bilateral lesion distribution resulting from multifocal microthrombi in the branches of the middle or rostral cerebral arteries that supply the prefrontal cortex and olfactory lobe (Figure 1A). Despite 3–4 days of generalized seizures, there was no evidence of self-inflicted trauma to the skull.

Histopathological features of the ischemic infarct, characterized by liquefactive necrosis with a central hemorrhagic lesion, neuronal degeneration, and spongiosis at the periphery, were consistent with established canine ischemic brain findings (17, 18). However, the peri-infarct or penumbral zone was hypercellular and displayed histological features resembling those previously reported in canine structural epilepsy. These features include an increase in new vessels, microglial cells, and astrocytes, along with swollen oligodendroglial cells and ischemic neurons. Consequently, these peri-infarct cellular areas were examined for ECM changes to understand the lesion’s age and for VEGF activity to evaluate the lesion’s epileptogenic nature.

Regarding ECM-related changes, we found a reticulin reaction in the neovascular area and intercellular spaces. ECM components, such as reticulin, are used in brain pathology studies, including those examining active canine distemper lesions and differentiating brain tumors during diagnosis (19, 20). Furthermore, reticulin is an early marker of inflammation, appearing within 2–3 days, as demonstrated in skin injury model studies (21). Conversely, late-appearing ECM markers of inflammation, such as mature collagen and vascular elastin, were weak or completely absent. This absence ruled out the presence of slow-progressing extracellular reactions, such as astrocytic gliosis, which is a potential pre-epileptic focus reported in structural epilepsy studies involving Beagles and Shetland Sheepdogs (8).

A key histological feature of the pre-epileptic lesion is vascular proliferation, characterized by increased microvessels expressing von Willebrand factor (vWF) and VEGF (13, 14). Under hypoxic conditions, hypoxia-inducible factor (HIF) upregulates VEGF, thereby stimulating angiogenesis (14). While chronic hypoxia has been associated with extramedullary hematopoiesis in the choroid plexus in 35% of seizure-prone male Beagle dogs, this condition was absent in the examined animal (22). Instead, we observed an immediate and significant increase in new vessel growth within the peri-infarct region, resembling proliferative benign changes in the central nervous system, which was not included in our initial differential diagnosis (23). Furthermore, proliferating vessels in temporal lobe epilepsy can activate recurrent seizures, and the same condition in ischemia can cause post-stroke epilepsy (24). However, several factors did not favor an ischemic cause in this study: the absence of underlying conditions for stroke in the animal and the observation of vascular growth older than 3 days in the penumbral infarct zone, which is less likely to occur during a 5-day seizure episode (25). Moreover, the absence of hemosiderin-laden macrophages indicates a recent hemorrhage. Therefore, a pre-existing epileptic focus is more likely; however, late extracellular matrix changes in collagen or elastin, which would have precluded the confirmation of gliosis, were absent. Nonetheless, the vascular growth was striking, along with VEGF expression from both endothelial and surrounding glial cells, which is a more likely trigger for the epileptic seizure (Figure 2H). Upregulated VEGF was found in newly formed vessels after the seizure. Although the increase in VEGF expression is neuroprotective by increasing angiogenesis, they also incite neuroinflammation, impair the blood–brain barrier (BBB), cause albumin leakage, and form gliosis. Over the 5-day period, we observed a 30% decline in serum albumin levels while globulin levels remained relatively steady; this selective loss is likely attributed to neuroinflammation-driven vascular leakage. This neuroinflammatory profile was further reflected in a marked absolute monocytosis (0.5 to 15 × 10^3^/μL). This shift is consistent with leukotriene-driven myeloid recruitment and the simultaneous decrease in neutrophils previously reported in the canine pentylenetetrazole-induced seizure model (26). The hemorrhage observed in the brain sample is a consequence of BBB impairment resulting from the seizures (12, 23). Similar to hemorrhage, another possible vascular event after the onset of the seizure was multiple thrombi, given that the platelet count was normal on the first day of the episode. The hemorrhage was certainly not trauma-induced, as there was an absence of widespread edema or axonal injury (27). In summary, the initial seizures most likely triggered hemorrhage and thrombus, and the resulting hypoxia induced peripheral vascular growth, further inducing post-stroke epileptic seizures.

Several limitations remain in this study, most notably the absence of advanced imaging during the early clinical course. Such imaging, performed prior to infarct formation, might have definitively identified an underlying idiopathic epilepsy before the progression became structural. Furthermore, we did not conduct a definitive differentiation between structural changes arising from a pre-existing epileptic focus and those that were purely induced by the stroke. The inclusion of additional immunohistochemical markers could have provided deeper insights. For instance, GFAP could be used to evaluate early astrocytic gliosis; VEGFR-1 to assess exclusive endothelial expression; VEGFR-2, to assess its role in the protective upregulation of neuronal signaling following seizures; albumin staining to detect vascular leakage; and Iba1 to characterize activated microglia involved in neuroinflammation (28, 29).

Due to the animal’s death, a longitudinal follow-up was not possible, leaving this as a single report on the dual pathology of stroke and seizure in a dog. Nevertheless, this case offers a compelling animal model for post-stroke epilepsy (PSE). It suggests that early and late PSE might be better distinguished by specific cortical changes rather than by duration alone. Specifically, cortical VEGF expression coupled with extensive vascular growth may serve as a reliable tissue-based diagnostic marker, comparable to the use of hemorrhage-derived iron in clinical imaging (11). From a therapeutic perspective, these findings are noteworthy, as anti-VEGF interventions have already been shown to reduce stroke-induced brain swelling and edema in other animal models (30).

Our study demonstrates that the onset of acute structural epilepsy is associated with significant vascular proliferation. These developments appear essential for seizure induction, continue to persist in chronic stages, and likely serve as a recurring source of seizure activity in affected dogs (14). Ultimately, the occurrence of subsequent seizures following the hemorrhage gives this case a distinct characteristic of early-stage post-stroke epilepsy. The clinical progression, combined with the observed vascular lesions and widespread VEGF expression, provides valuable insights into the complex pathology of both epilepsy and post-stroke epileptic seizures in the canine population.

## Data Availability

The original contributions presented in the study are included in the article/Supplementary material, further inquiries can be directed to the corresponding author.

## References

[1] Knowles K. Idiopathic epilepsy. Clin Tech Small Anim Pract. (1998) 13:144–51. doi: 10.1016/S1096-2867(98)80035-2 9775504

[2] Ekenstedt KJ Oberbauer AM. Inherited epilepsy in dogs. Top Companion Anim Med. (2013) 28:51–8. doi: 10.1053/j.tcam.2013.07.001, 24070682

[3] Erlen A Potschka H Volk HA. Seizure occurrence in dogs under primary veterinary care in the UK: prevalence and risk factors. J Vet Intern Med. (2018) 32:1665–76. doi: 10.1111/jvim.15290, 30216557 PMC6189390

[4] Löscher W. Dogs as a natural animal model of epilepsy. Front Vet Sci. (2022) 9:928009. doi: 10.3389/fvets.2022.928009, 35812852 PMC9257283

[5] Blades Golubovic S Rossmeisl JH. Status epilepticus in dogs and cats, part 1: etiopathogenesis, epidemiology, and diagnosis. J Vet Emerg Crit Care. (2017) 27:278–87. doi: 10.1111/vec.12605, 28445615

[6] Wessmann A Chandler K Garosi L. Ischaemic and haemorrhagic stroke in the dog. Vet J. (2009) 180:290–303. doi: 10.1016/j.tvjl.2007.12.023 18579421

[7] Garosi L McConnell JE Platt SR Barone G Baron JC de Lahunta A . Results of diagnostic investigations and long-term outcome of 33 dogs with brain infarction (2000–2004). J Vet Intern Med. (2005) 19:725–31. doi: 10.1111/j.1939-1676.2005.tb02752.xv 16231718

[8] Danciu C Gonçalves R Caldero CJ Danciu C-G Posporis C Espinosa J . Comorbidities, long-term outcome and poststroke epilepsy associated with ischemic stroke: a multicenter observational study of 125 dogs. J Vet Intern Med. (2025) 39:e17291. doi: 10.1111/jvim.17291, 39711420 PMC11664234

[9] Shin SHS Moon KR Kim EY Rho YI. Two cases of disseminated intravascular coagulation due to status epilepticus with high fever. J Korean Pediatr Soc. (2001) 44:1062–5. Available online at: https://www.ecep.org/journal/view.php?number=2001440913

[10] Dunham J Horridge M Lim JH Lyons BM Wiggen K. Case report: naturally occurring neurogenic stunned myocardium in a dog secondary to status epilepticus. Front Vet Sci. (2024) 11:1376107. doi: 10.3389/fvets.2024.1376107, 38895716 PMC11185869

[11] Tanaka T Ihara M Fukuma K Mishra NK Koepp MJ Guekht A . Pathophysiology, diagnosis, prognosis, and prevention of poststroke epilepsy: clinical and research implications. Neurology. (2024) 102:e209450. doi: 10.1212/WNL.0000000000209450, 38759128 PMC11175639

[12] Larsson D Farahmand B Åsberg S Zelano J. Risk of stroke after new-onset seizures. Seizure. (2020) 83:76–82. doi: 10.1016/j.seizure.2020.09.033, 33120325

[13] Montgomery DL Lee AC. Brain damage in the epileptic Beagle dog. Vet Pathol. (1983) 20:160–9. doi: 10.1177/030098588302000203, 6836872

[14] Sakurai M Morita T Takeuchi T Shimada A. Relationship of angiogenesis and microglial activation to seizure-induced neuronal death in the cerebral cortex of Shetland sheepdogs with familial epilepsy. Am J Vet Res. (2013) 74:763–70. doi: 10.2460/ajvr.74.5.763, 23627390

[15] Palazzi X Pardo ID Ritenour H. A technical guide to sampling the Beagle dog nervous system for general toxicity and neurotoxicity studies. Toxicol Pathol. (2022) 50:432–65. doi: 10.1177/01926233221099300, 35730663

[16] Jankauskas M Gradeckienė A Čižinauskas S. Extended analysis of status epilepticus and cluster seizures in dogs in the context of overall epilepsy incidence: 254 cases. Animals (Basel). (2025) 15:2807. doi: 10.3390/ani15192807, 41096402 PMC12524279

[17] Kang MH Jeong WP Nam CS Yoon JW Choi DM Lee GS . Case report: ischemic brain infarction and cognitive dysfunction syndrome in an aged dog. Front Vet Sci. (2025) 12:1563798. doi: 10.3389/fvets.2025.1563798, 40177667 PMC11963772

[18] Jeon JH Jung HW Jang HM Moon JH Park KT Lee HC . Canine model of ischemic stroke with permanent middle cerebral artery occlusion: clinical features, MRI, histopathology, and immunohistochemistry. J Vet Sci. (2015) 16:75–85. doi: 10.4142/jvs.2015.16.1.75 25269716 PMC4367152

[19] Oliveira LA Viscone EA Medeiros-Ronchi AA Bandarra MB. Histopathological features of the brain extracellular matrix from dogs with distemper. Arq Bras Med Vet Zootec. (2023) 75:831–8. doi: 10.1590/1678-4162-12651

[20] Awadalla AS Al Essa AM Al Ahmadi HH Al Ojan A Muazen Y Alsayyah A . Gliosarcoma case report and review of the literature. Pan Afr Med J. (2020) 35:26. 10.11604/pamj.2020.35.26.17577 PMC7170742 32341747

[21] Betz P Nerlich A Wilske J. Immunohistochemical localization of collagen type III and V for estimation of wound age. Int J Legal Med. (1993) 105:329–32. doi: 10.1007/BF01222117 8518198

[22] Woicke J Al-Haddawi MM Bienvenu JG Caverly Rae JM Chanut FJ Colman K . International harmonization of nomenclature and diagnostic criteria (Inhand): nonproliferative and proliferative lesions of the dog. Toxicol Pathol. (2021) 49:33393871:5–109. doi: 10.1177/0192623320968181 33393871

[23] Marr J Miranda IC Miller AD Summers BA. A review of proliferative vascular disorders of the CNS in animals. Vet Pathol. (2021) 58:864–80. doi: 10.1177/0300985820980707 33302811

[24] Rigau V Morin M Rousset MC de Bock F Lebrun A Coubes P . Angiogenesis is associated with blood–brain barrier permeability in temporal lobe epilepsy. Brain. (2007) 130:1942–56. doi: 10.1093/brain/awm118, 17533168

[25] Mărgăritescu O Mogoantă L Pirici I Pirici D Cernea D Mărgăritescu CL. Histopathological changes in acute ischemic stroke. Romanian J Morphol Embryol. (2009) 50:327–39. Available online at: https://europepmc.org/article/med/19690757 19690757

[26] Koo Y Yun T Chae Y Lee D Kim H Yang MP . Evaluation of the covariation between leukotriene B4, prostaglandin E2, and hematologic inflammatory parameters in a canine pentylenetetrazole-induced seizure model. Front Neurosci. (2024) 18:1451902. doi: 10.3389/fnins.2024.1451902, 39723425 PMC11668773

[27] Crawford AH Beltran E Danciu C Yaffy D. Clinical presentation, diagnosis, treatment and outcome in animals with global hypoxic–ischemic brain injury. J Vet Intern Med. (2023) 37:1428–37. doi: 10.1111/jvim.16790, 37316975 PMC10365066

[28] Croll SD Goodman JH Scharfman HE. Vascular endothelial growth factor (VEGF) in seizures: a double-edged sword. Adv Exp Med Biol. (2004) 548:57–68. doi: 10.1007/978-1-4757-6376-8_4, 15250585 PMC2504497

[29] Vezzani A. VEGF and seizures: cross-talk between endothelial and neuronal environments. Epilepsy Curr. (2005) 5:72–4. doi: 10.1111/j.1535-7597.2005.05209.x, 16059441 PMC1176313

[30] Kim ID Cave JW Cho S. Aflibercept, a VEGF (Vascular Endothelial Growth Factor) trap, reduces vascular permeability and stroke-induced Brain swelling in obese Mice’. Stroke. (2021) 52:2367–48. doi: 10.1161/STROKEAHA.121.034362, 34192895 PMC8312568

