# Supplementary material for: Case Report: Post-stroke hemorrhagic infarction in a status epilepticus Beagle dog
Source: Front Vet Sci. 2026 Mar 25;13:1764817. doi: 10.3389/fvets.2026.1764817 (PMC13059657; doi:10.3389/fvets.2026.1764817)
Supplement: Supplementary file 1 [file Table_1.docx]

# Supplementary Table 1

**Timeline of the Case**

| **Symptomatic period** | **Symptoms (prominent)** | **MGCS score** | **Treatment** | **Comment (Diagnosis/ Veterinary Care)** |
| --- | --- | --- | --- | --- |
| Day 0 | - Body weight: 4.1kg - Acute onset - Cluster Seizures - Stretching of legs - Foaming saliva - Conjunctival hyperemia. | 6  (Grave) | - Diazepam IV bolus to stop Status Epilepticus (SE); - 0.9% Normal Saline-Dextrose supplemented with 5% Dextrose (NS-D) for inappetence and hypoglycemia - Meloxicam to reduce fever (0.2mg/kg- IV) | - Reactive Seizures (Toxic/Metabolic?) - High Creatinine Kinase & Low Glucose (Muscle contractions) - Animal moved to an isolation unit - Wet towel application - Salivation cleared constantly to avoid aspiration - Animal placed elevated in lateral recumbency and in foam bedding on the floor |
| Day 1 | - Cluster Seizures - Increased frequency of seizures/SE - Intermittent limb jerks | 9  (Guarded) | - Potassium Bromide (KBr) - Loading dose (600mg/kg b.w, *per os*) - NS-D (IV) - Diazepam Continuous infusion 0.5mg/kg b.w | - Only one loading dose of KBr could be administered orally due to difficulty in intubation/jerky jaw movements - Failure to regain consciousness between seizures noticed - Animal rotated to avoid bed sore. |
| Day 2 | - Persistent unconsciousness - No response to stimuli. | 7  (Grave) | - KBr – Maintenance 100mg/kg b.w (2 doses) - NSD (IV) - Meloxicam (0.2mg/kg- SC) | - Non-Responsive Status – Increased intracranial pressure suspected. |
| Day 3 | - Stupor - Limb stiffness. | 5  (Grave) | - 7.5% Hypertonic Saline followed by NS-D (IV) fluids | - Terminal Body weight: 3.2 kg - Cachexia - No improved consciousness to hypertonic saline |
| Day 4 | - Comatose, moribund - No response to noxious stimuli. | 3  (Grave) |  |  |

MGCS- Modified Glasgow Coma Scale; IV- Intra-venous; SC- Subcutaneous; b.w- body weight
