# Supplementary material for: Case Report: Post-stroke hemorrhagic infarction in a status epilepticus Beagle dog
Source: Front Vet Sci. 2026 Mar 25;13:1764817. doi: 10.3389/fvets.2026.1764817 (PMC13059657; doi:10.3389/fvets.2026.1764817)
Supplement: Supplementary file 2 [file Table_2.docx]

Supplementary Material

# Supplementary Table 2. Initial and terminal, complete blood count and serum chemistry results of the male Beagle dog

| **Parameters** | **Unit** | **Reference range of Beagles** | | | **Day0** | **Day5** |  | **Parameters** | **Unit** | **Reference range of Beagles** | | | **Day0** | **Day5** |
| --- | --- | --- | --- | --- | --- | --- | --- | --- | --- | --- | --- | --- | --- | --- |
|  |  | Upto 24m^$^ | N | 24-60m^#^ |  |  |  |  |  | Upto 24m^$^ | N | 24-60m^#^ |  |  |
| **RBC** | 10^6^/µL | 5.98-8.14 | 181 | 8.09 ± 0.4 | 7.96 | 6.25 |  | **Total Protein** | g/dL | 5.3-7.3 | 228 | 7.15 ± 0.4 | 7.0 | 5.7↓ |
| **HCT** | % | 40.3-8.14 | 181 | 7.15 ± 0.4 | 56.8 | 46.8 |  | **Albumin** | g/dL | 2.7-4.2 | 227 | 3.29 ± 0.2 | 3.5 | 2.3↓ |
| **HGB** | g/dL | 13.0-18.3 | 181 | 17.9 ± 1.1 | 18.8 | 14.3 |  | **Globulin** | g/dL | 2.0-3.5 | 227 | - | 3.1 | 2.9 |
| **MCV** | fL | 62.5-72.4 | 181 | 65.7 ± 3.0 | 71.3 | 74.9 |  | **Glucose** | mg/dL | 73.2-112.9 | 228 | 86.8±4.5 | 52.3↓ | 110.3 |
| **MCH** | pg | 20.3-24.1 | 179 | 22.1 ± 1.0 | 23.6 | 22.9 |  | **ALT** | U/L | 16.6-59.8 | 222 | 64.9 ± 64.0 | 112.9 | 129.9 |
| **MCHC** | g/dL | 31.3-34.0 | 180 | 33.7± 0.4 | 33.1 | 30.6 |  | **AST** | U/L | 20.3-45.8 | 224 | 40.1 ± 7.9 | 404.4↑ | 272.9 |
| **WBC** | 10^3^/µL | 5.53-13.53 | 180 | 8.01 ± 1.81 | 8.07 | 18.91 |  | **ALP** | U/L | 52.2-287.4 | 227 | 188.2 ± 100.8 | 369.5 | 475.2 |
| **NEU** | % | 47.8-74.4 | 157 | - | 83.1 | 2.6↓ |  | **BUN** | mg/dL | 6.9-14.7 | 119 | 14.28±2.5 | 21.2↑ | 28.8 |
| **LYM** | % | 17.9-43.1 | 158 | - | 8.5 | 14.1 |  | **Creatinine** | mg/dL | 0.40-0.98 | 228 | 0.66 ±0.07 | 1.06↑ | 0.83↓ |
| **MONO** | % | 2.6-7.7 | 158 | - | 6.1 | 80.9↑ |  | **BUN/CRE** | - | - | - | - | 20.0: 1 | 34.7: 1↑ |
| **EOS** | % | 0.9-6.9 | 156 | - | 0.1 | 0.0 |  | **CK** | U/L | 98-541 | 223 | 171.3 ± 68.9 | 9966↑ | 13947↑ |
| **BASO** | % | 0.1-0.8 | 157 | - | 1.1 | 0.3 |  | **Na** | mmol/L | 142-151 | 208 | 150.4 ± 1.2 | 149 | 168 |
| **PLT** | 10^3^/µL | 300-754 | 181 | 261 ± 71 | 439 | 290↓ |  | **K** | mmol/L | 4.23-5.37 | 206 | 4.8 ± 0.2 | 5.15 | 4.97 |
| **NEU** | 10^3^/µL | 3.14-9.04 | 179 | - | 6.71 | 0.49↓ |  | **Cl** | mmol/L | 105.1-115.7 | 208 | 113.5 ± 1.4 | 121.1 | 139.7 |
| **LYM** | 10^3^/µL | 1.24-3.98 | 179 | - | 0.68 | 2.66 |  | **PHOS** | mg/dL | 2.51-6.23 | 207 | - | 3.41 | 4.96 |
| **MONO** | 10^3^/µL | 0.18-0.71 | 178 | - | 0.49 | 15.3↑ |  | ^$^**Upto 24month age – In-house data of Zydus Research Center, Zydus Life Sciences Limited.**  ^#^**24–60-month age – Published data of 15 male Beagle dogs from** *Animal Model Exp Med. 2018 Dec 5;1(4):282–294* | | | | | | |
| **EOS** | 10^3^/µL | 0.09-0.56 | 176 | - | 0.01 | 0.01 |  |  |  |  |  |  |  |  |
| **BASO** | 10^3^/µL | 0.01-0.06 | 179 |  | 0.09 | 0.06 |  |  |  |  |  |  |  |  |
