# Supplementary material for: Case Report: Post-stroke hemorrhagic infarction in a status epilepticus Beagle dog
Source: Front Vet Sci. 2026 Mar 25;13:1764817. doi: 10.3389/fvets.2026.1764817 (PMC13059657; doi:10.3389/fvets.2026.1764817)
Supplement: Supplementary file 3 [file Table_3.docx]

Supplementary Material

**Supplementary Figures**

S1: Additional view of the necrotic foci in frontal lobe showing loss of gyri &sulci (arrow)

S2: Carcass of the sacrificed animal showing severe body weight loss

S3: Rectum of the animal after potassium bromide treatment shows brown colored content which also smears the grossing table

S4: Neuroinflammation involving reactive astrocytes (arrow) in 40x magnification. Hematoxylin and Eosin (HE)

S5: Neuroinflammation involving swollen oligodendroglial (arrows) and minimal microglial cells in 40x magnification. Hematoxylin and Eosin (HE)

S6: Bright-field image in 100x magnification of vessel showing VEGF-A positive fluorescence in Fig.2f

S7: Additional site of crowded vascular proliferation (yellow arrow) at a distance from a small area of necrotic center (black arrow) in a 4x magnification. Inset shows a 40x magnification of yellow arrow marked area showing hypercellular area with proliferating endothelial and glial cells

S8: Polymorphonuclear cells lining inside the blood vessel in necro-hemorrhagic cortical area (HE) at 40x magnification

S9: Higher magnification of choroid vessels in the sulcus of the frontal lobe involved in ischemic cortical changes.
