# Supplementary figures and images for: Case Report: Post-stroke hemorrhagic infarction in a status epilepticus Beagle dog
Source: Front Vet Sci. 2026 Mar 25;13:1764817. doi: 10.3389/fvets.2026.1764817 (PMC13059657; doi:10.3389/fvets.2026.1764817)

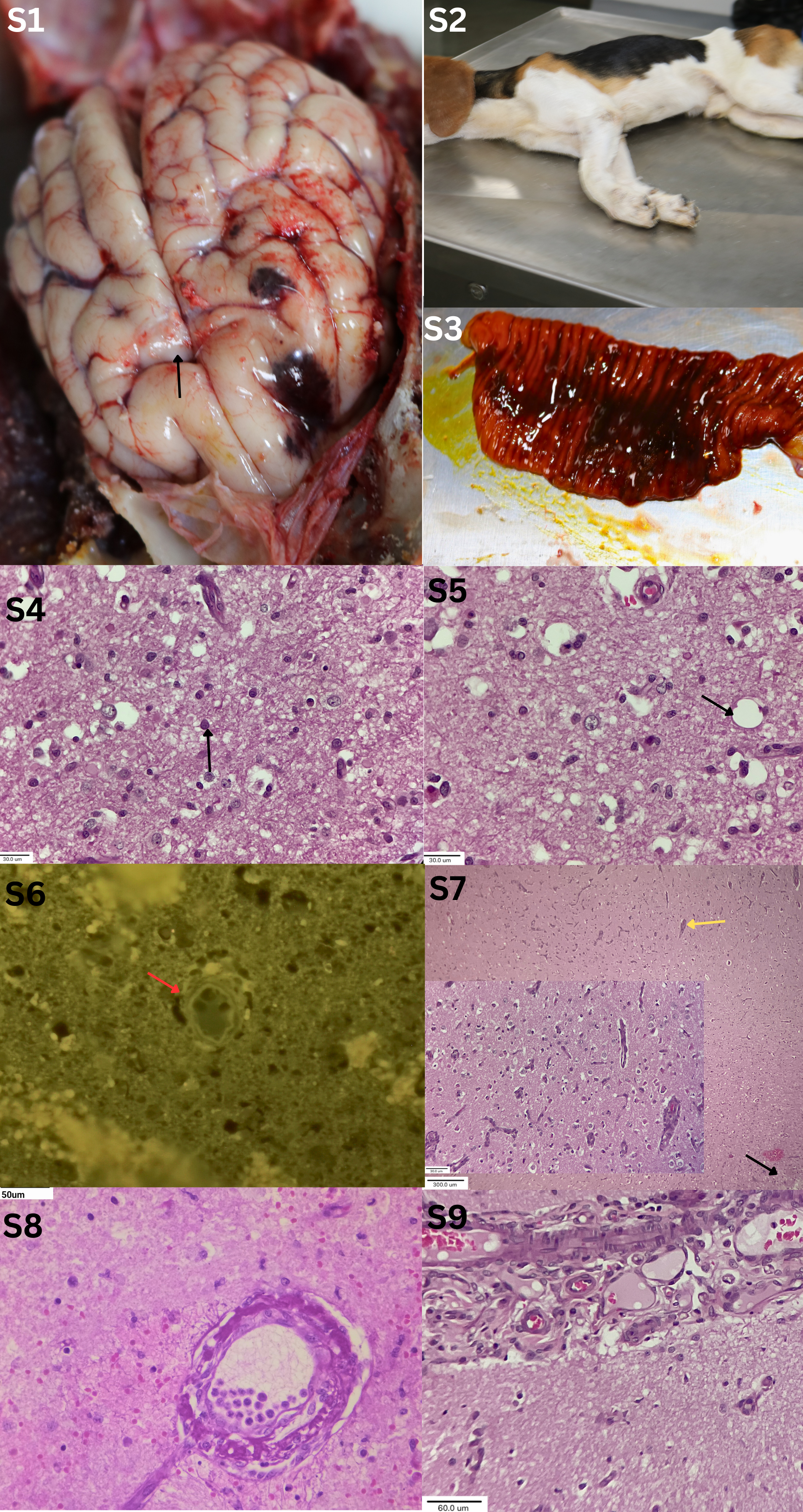

Supplement: Supplementary file 4 [file Image_1.TIFF]
